# Supplementary material for: Investigating the impact of paternal aging on murine sperm miRNA profiles and their potential link to autism spectrum disorder
Source: Sci Rep. 2023 Dec 7;13:20608. doi: 10.1038/s41598-023-47878-z (PMC10703820; doi:10.1038/s41598-023-47878-z)
Supplement: Supplementary file 1 — Supplementary Figures. [file 41598_2023_47878_MOESM1_ESM.docx]

**Supplementary Figures**

**Title: Investigating the impact of paternal aging on murine sperm miRNA profiles and their potential link to autism spectrum disorder**

Kazusa Miyahara^+^, Misako Tatehana^+^, Takako Kikkawa, and Noriko Osumi*

Department of Developmental Neuroscience, Center for Advanced Research and Translational Medicine (ART), Tohoku University Graduate School of Medicine, Sendai, Japan.

^+^These authors contributed equally to this work

*Correspondence to: Noriko Osumi, PhD

Department of Developmental Neuroscience, Center for Advanced Research and Translational Medicine (ART), Tohoku University School of Medicine, Sendai, Japan.

TEL: 022-717-8201; FAX: 022-717-8205

E-mail: osumi＠med.tohoku.ac.jp

**
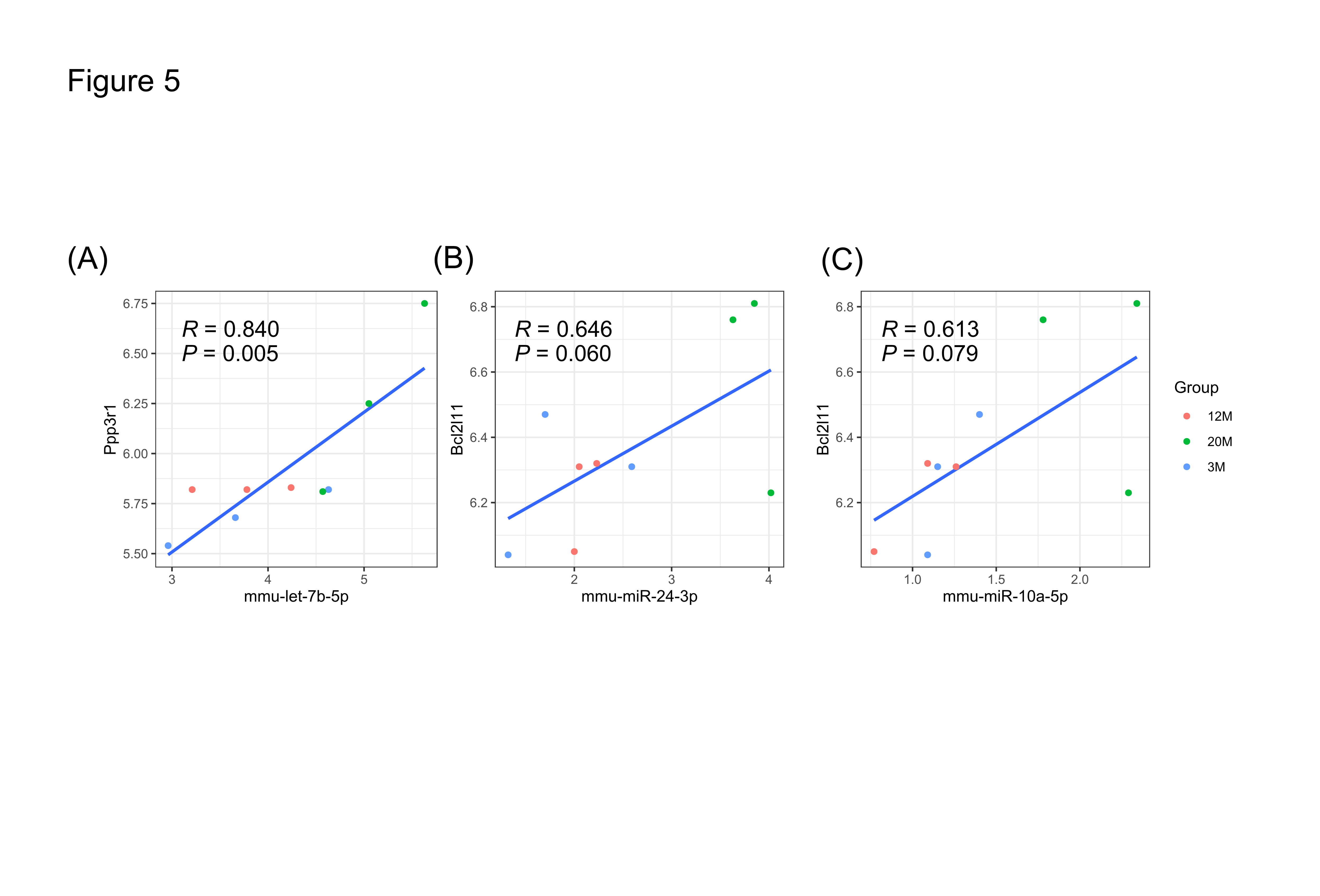
Supplementary Figure S1**

**Supplementary Figure S1.** Correlation analysis between apoptosis-related genes, *Ppp3r1* and *Bcl2l11*, and their regulator miRNAs. Scatter plot shows the correlation between *Ppp3r1* and *mmu-let-7b-5p*(**A**), *Bcl2l11* and *mmu-miR-10a-5p* (**B**), and *Bcl2l11* and *mmu-miR-24-3p* (**C**).

**
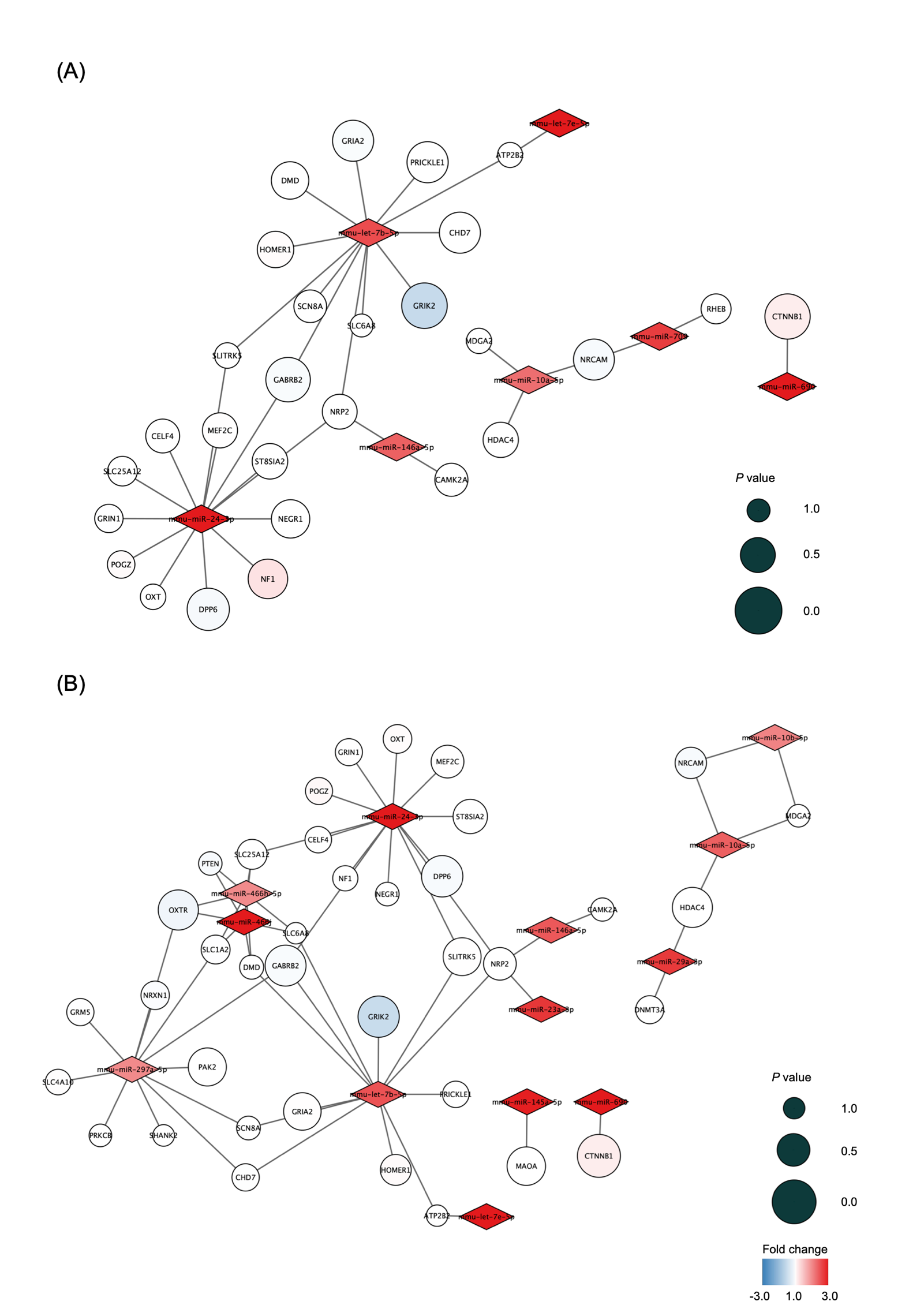
Supplementary Figure S2**

**Supplementary Figure S2.** Network of miRNAs and SFARI genes. Network of significantly altered miRNAs and their target autism-related SFARI genes when comparing 3M vs 20M (**A**) and 12M vs 20M (**B**). Node color indicates the fold change of the miRNA and target gene expression. Node size indicates the significance of the differential expression; larger sizes indicate smaller *P* values.
